# Microbiome composition and presence of cultivable commensal groups of Southern Tamanduas (*Tamandua tetradactyla*) varies with captive conditions

**DOI:** 10.1186/s42523-024-00311-w

**Published:** 2024-05-02

**Authors:** Ahmad Amin, Chahrazed Mekadim, Nikol Modrackova, Petra Bolechova, Jakub Mrazek, Vera Neuzil-Bunesova

**Affiliations:** 1 Department of Microbiology, Nutrition and Dietetics, Faculty of Agrobiology, Food and Natural Resources, Czech University of Life Sciences Prague, Kamycka 129, 165 00 Prague 6, Czech Republic; 2 Institute of Animal Physiology and Genetics, The Czech Academy of Sciences, v. v. i., Videnska 1083, 142 20 Prague, Czech Republic; 3 Department of Ethology and Companion Animal Science, Faculty of Agrobiology, Food and Natural Resources, Czech University of Life Sciences, Kamycka 129, 165 00 Prague 6, Czech Republic

**Keywords:** Anteater, Fecal microbiome, Cultivable microbes, Captive environment, Diet

## Abstract

Southern Tamanduas (*Tamandua tetradactyla*) belong to the specialized placental myrmecophages. There is not much information about their intestinal microbiome. Moreover, due to their food specialization, it is difficult to create an adequate diet under breeding conditions. Therefore, we used 16S rDNA amplicon sequencing to analyze the fecal microbiome of captive Southern Tamanduas from four locations in the Czech Republic and evaluated the impact of the incoming diet and facility conditions on microbiome composition. Together with the microbiome analysis, we also quantified and identified cultivable commensals. The anteater fecal microbiome was dominated by the phyla Bacillota and Bacteroidota, while Pseudomonadota, Spirochaetota, and Actinobacteriota were less abundant. At the taxonomic family level, *Lachnospiraceae, Prevotellaceae, Bacteroidaceae, Oscillospiraceae, Erysipelotrichaceae, Spirochaetaceae, Ruminococcaceae, Leuconostocaceae*, and *Streptococcaceae* were mainly represented in the fecal microbiome of animals from all locations. Interestingly, *Lactobacillaceae* dominated in the location with a zoo-made diet. These animals also had significantly lower diversity of gut microbiome in comparison with animals from other locations fed mainly with a complete commercial diet. Moreover, captive conditions of analyzed anteater included other factors such as the enrichment of the diet with insect-based products, probiotic interventions, the presence of other animals in the exposure, which can potentially affect the composition of the microbiome and cultivable microbes. In total, 63 bacterial species from beneficial commensal to opportunistic pathogen were isolated and identified using MALDI-TOF MS in the set of more than one thousand selected isolates. Half of the detected species were present in the fecal microbiota of most animals, the rest varied across animals and locations.

**Supplementary Information:**

The online version contains supplementary material available at 10.1186/s42523-024-00311-w.

## Background

Anteaters belong to the order Pilosa comprising two families: *Cylopedidae* with a single species representative, the Silk Anteater (*Cyclopes didactylus*), and *Myrmecophagidae* with three species representatives including the Northern Tamandua (*Tamandua mexicana*), the Southern Tamandua (*Tamandua tetradactyla*), and the Giant Anteater (*Myrmecophaga tridactyla*) [1]. They are fascinating placental mammals known as myrmecophagous (ant- and termite-eating) and represent an excellent example of evolutionary convergence driven by extreme diet specialization [2], and possessing a unique digestive system. Anteaters have a longer small intestine, suggesting slower digestion than other animals of the same order, adapted to extract nutrients from insect prey more efficiently. Moreover, their saliva contains enzymes that aid in the breakdown of insect exoskeletons. Furthermore, as hydrochloric acid is not produced in their stomachs like in most mammals, they depend on the supply of formic acid from swallowed ants for their digestion. Additionally, anteaters have a large cecum, a specialized pouch connected to the large intestine [3, 4]. To utilize chitin, cellulose, and other complex carbohydrates, they live in mutually beneficial symbiosis with their gut microbiota that helps them break down these substrates [5]. The anteater diet primarily consists of ants and termites, including their developmental stages, although they may occasionally consume other insects or insect larvae [6]. Moreover, their feeding habits depend on location and time of season [7]. On a larger taxonomic scale, the gut microbiome has changed drastically in mammals living in the same feeding habitats; diet seems to be a key driving factor [8]. In captivity, anteaters are either fed a complete commercial diet, which nutritionally mimics their natural diet, or a zoo-made diet that is often composed of a variety of ingredients such as a raw meat, milk products, eggs, cereals, dog pellets, fruits, clay, multivitamins, and trace mineral supplements [4, 9].

Recent studies increasingly note the effects of captivity and the facility environment on the anteater’s microbiome [8–11]. Diet changes, treatment, and reduced contact with other individuals, species, and variable environmental substrates affect bacterial diversity [10]. As commensal microbes are essential to many aspects, including animal health, it is important to understand how captive differences can affect the host gut microbiome. However, the number of studies dealing anteater microbiome composition is still limited, and mainly available for the Giant Anteater [2, 12, 13].

Our study aimed to analyze the fecal microbiome of captive Collared Anteaters (*Tamandua tetradactyla*) from four locations in the Czech Republic and to evaluate the impact of the provided diet and facility conditions on their fecal microbiome. Along with a microbiome analysis to quantify and identify cultivable commensals such as bifidobacteria, clostridia, coliforms, lactobacilli, and other lactic acid bacteria.

## Methods

### Ethical statement and feces sampling

Animal feces were sampled during routine daily maintenance at zoos and from a private breeder (Czech Republic). Zoological institutions have rigorous standards for animal welfare and are accredited by the European Association of Zoos and Aquaria. All procedures involving animals adhered to recommendations of the “Guide for the Care and Use of Animals” by the Czech University of Life Sciences Prague and complied with the European Directive 2010/63/EU.

The following criteria were set for the selection of animals for the study: having at least two adult individuals from the given location of both sexes, taking samples at the same period of the year, having three samples per individual with an approximate weekly sampling interval, considering the digestion length and the defecation frequency. In total, samples were obtained from 11 Southern Tamanduas (AE1–11; *Tamandua tetradactyla*). The animals were kept at 4 different locations (L1–4) in the Czech Republic (Zoo Olomouc, Zoo Lešná, Zoo Ústí nad Labem, and Javornice from a private breeder). Information on animal age and sex, diet, exposure sharing, and sampling date is shown in Table 1. For each animal, 3 samples were collected and analyzed at weekly intervals.

**Table 1:** The list of anteaters, including locations, captive conditions, and intervals of fecal sampling

Location	Animal	Sex	Birthdate	Contact with other animals	Diet	Probiotic supplements	Date of fecal sample (FS) collection		
							FS1	FS2	FS3
Zoo Olomouc (**L1**)	**AE1**	Female	26.10.2016	Two-toed sloth, wild guinea pig	Zoo made diet^A^	NutriMix Probiotic^C^	09.06.2022	15.06.2022	23.06.2022
	**AE2**	Female	25.07.2020				09.06.2022	15.06.2022	23.06.2022
	**AE3**	Male	02.05.2010	Squirrel monkey, chacoan mara			09.06.2022	15.06.2022	23.06.2022
	**AE4**	Female	30.01.2022	Two-toed sloth, wild guinea pig			09.06.2022	15.06.2022	23.06.2022
	**AE5**	Female	15.02.2022				09.06.2022	02.07.2022	08.08.2022
Private breeder Javornice (**L2**)	**AE6**	Male	ND	Not	Granovit^B^	not	19.08.2022	23.08.2022	29.08.2022
	**AE7**	Female	ND				19.08.2022	23.08.2022	29.08.2022
Zoo Ústí nad Labem (**L3**)	**AE8**	Male	01.01.2022	White-faced sakis	Granovit^B*^	Fortiflora Canine Probiotic^D^	17.06.2022	30.06.2022	12.07.2022
	**AE9**	Male	01.01.2022				20.06.2022	27.06.2022	08.07.2022
Zoo Lešná (**L4**)	**AE10**	Male	ND	Geoffroy’s spider monkey	Granovit^B**^	not	18.07.2022	20.07.2022	29.07.2022
	**AE11**	Female	ND				18.07.2022	20.07.2022	25.07.2022

ND—no data, L—location, AE—animal host (anteater)

^A^Lean poultry meat, bananas, quail eggs, Luvos (HEILERDE, Germany), dog pellets—Sensitive Lamb & Rice Mini (Fortify, Czech Republic), honey, formic acid, Supradyn CoQ10 energy (Bayer, Germany), Kanavit (Zentiva, Czech Republic), taurin (fa Trouw Nutrition, Biofactory), honeycombs—according to the current health condition of the animals, as well as eggs, grapefruit, and tomato additionally provided for females and pups (AE1,2,4, and 5)

^B^Complete commercial diet/Granovit—Insectivore with insect meal (Granovit Zoofeed, AG, Switzerland)—Insect protein meal, poultry meat meal, potato protein, apple pomace, oat flakes, sugar, poultry fat, corn, corn gluten meal, cellulose, soybean oil, wheat germs, mineral and trace-element premix, calcium phosphate, shrimp shells, fish oil, fructooligosaccharides, inulin, salt, minerals, formic acid, *Enterococcus faecium* E 1708

^C^NutriMix Probiotic (fa Trouw Nutrition, Biofaktory)—*Saccharomyces cerevisiae* CNCM I-1077, *Bacillus licheniformis* DSM 5749, *Bacillus subtilis* DSM 5750, *Enterococcus faecium* NCIMB 10415

^D^Fortiflora Canine Probiotic (Purina Pro Plan)—*Enterococcus faecium* SF68®

*Added honey, worker cells

**Added mealworms, boiled eggs, papaya, avocado

The bold indicates the samples/location description

### 16S rRNA gene amplicon sequencing

Total genomic DNA was extracted from 300 mg of fecal samples (FS; from three FS per one animal collected independently in intervals as shown in Table 1; 100 mg per each FS) using the QIAamp® PowerFecal® Pro DNA Kit (Qiagen, Germany) according to the manufacturer’s instructions. The extracted DNA was then used as a template for the preparation of amplicons from the V4 region of the 16S rRNA gene [14]. Libraries were prepared from the purified amplicons using the NEBNext Fast DNA Library Prep Set kit (New England Biolabs, USA), according to Milani et al. [15]. The sequencing was then performed on the Ion Torrent platform (Termo Fisher Scientifc, USA) as it was described previously by Mekadim et al. [16].

### Microbiome analyses

The obtained bacterial 16S rDNA sequences in FASTQ format were analyzed by QIIME 2 version 2022.2 pipeline [17]. Quality filtering of sequences and removal of chimaera were performed using the DADA2 [18] to obtain amplicons sequence variants (ASVs) using the method of denoise-pyro which denoises single-end sequences with trimming left of 15 and maximum length of 250. Mafft was used to align the sequences [19] and fasttree was used to construct a phylogenetic tree [20]. Then, ASVs were taxonomically classified using VSEARCH based on SILVA database (release 138) with a 99% threshold [15], query alignment coverage of 0.8 and maxaccepts and maxhits values were set at 1. The rarefaction was performed based on the sequence depth to normalize data. Alpha diversity was determined using Shannon and Simpson diversity indexes based on the Kruskal–Wallis test. Principal Coordinate Analysis (PCoA) was based on Bray–Curtis distance (beta diversity). The box plots for alpha diversity and the 2-dimensional PCoA plots were generated in R-Studio (http://www.rstudio.com/) using qiime2R (https://github.com/jbisanz/qiime2R) and ggplot2 (https://ggplot2.tidyverse.org) packages. Permutational multivariate analysis of variance (Adonis) and Bray–Curtis distance matrix were used to evaluate the dissimilarity among location groups with permutation set at 999. The linear discriminant analysis with effect size (LEfSe) algorithm was performed [21] in Galaxy module http://huttenhower.sph.harvard.edu/galaxy to identify bacterial families with significant differential relative abundances between location groups based on the factorial Kruskal–Wallis (KW) test and the pairwise Wilcoxon test with α value of 0.05 and threshold value of 2.0.

### Cultivation analysis of fecal samples

FS collection and cultivation analysis were performed according to Modrackova et al. [22] with some modifications. FS were collected in tubes containing a dilution buffer (5 g L^−1^ tryptone, 5 g L^−1^ nutrient broth No. 2, 2.5 g L^−1^ yeast extract (all Oxoid, Basingstoke, UK), 0.5 g L^−1^ L-cysteine, 1 mL L^−1^ Tween 80 (both Sigma-Aldrich, St. Louis, Missouri, USA), 30% glycerol (VWR, Radnor, Pennsylvania, USA), and glass pearls for homogenization. Media were prepared in an oxygen‐free carbon dioxide environment and then sterilized. After sampling, the tubes were stored at – 20 °C and were transported to the laboratory for analysis within 1 month. Decimal serial dilutions of FS were then spread on the following media. Wilkins–Chalgren Anaerobe Agar was supplemented with 5 g L^−1^ GMO-Free Soya Peptone (both Oxoid), 0.5 g L^−1^ L-cysteine, and 1 mL L^−1^ Tween 80 to determine total counts of anaerobic bacteria (WSP medium). Three variants of selective media were used for clostridial/bifidobacterial quantification and isolation. WSP-MUP: WSP agar supplemented with 100 mg L^−1^ of mupirocin (Oxoid) and 1 mL L^−1^ of acetic acid (Sigma-Aldrich); WSP-NORF 1: WSP agar supplemented with 100 mg L^−1^ of mupirocin, 100 mg L^−1^ of norfloxacin (both Oxoid), and 1 mL L^−1^ of acetic acid (Sigma-Aldrich); WSP-NORF 2: WSP agar supplemented with 100 mg L^−1^ of mupirocin, 200 mg L^−1^ of norfloxacin (both Oxoid), and 1 mL L^−1^ of acetic acid (Sigma-Aldrich). All plates were incubated anaerobically using GENbag anaer (bioMérieux, Craponne, France) at 37 °C for 2 days. Rogosa Agar (Oxoid) with acetic acid (1.32 mL L^−1^) and microaerophilic conditions were used for lactobacilli and other lactic acid bacteria at 37 °C for 48 h, and chromogenic Tryptone Bile X-Glucuronide medium (TBX; Oxoid) for *Escherichia coli* and other coliform bacteria at 37 °C for 24 h and under aerobic conditions.

Counts of bacterial colonies in log CFU g^−1^ within the four locations are shown as boxplots. The normality of data was evaluated by the Shapiro–Wilk W test (α = 0.05). Differences in bacterial counts on different cultivation media were assessed by a one-way ANOVA (α = 0.05) and Kruskal–Wallis test (α = 0.05) using STATISTICA software (StatSoft, Prague, Czech Republic) and Microsoft Office Professional Plus 2016 (Redmond, WA, USA).

### Colony isolation and identification

At least 6 isolates per each media and FS were collected; specifically, more than 108 isolates per each animal. The colonies were cultivated in tubes containing non-selective Wilkins-Chalgren Anaerobe Broth supplemented with 5 g L^−1^ GMO-Free Soya Peptone (both Oxoid, Basingstoke, UK), 0.5 g L^−1^ L-cysteine, and 1 mL L^−1^ Tween 80 (both Sigma-Aldrich, St. Louis, Missouri, USA) under anaerobic conditions [23] at 37 °C for 24 h. The purity was then checked by phase-contrast microscopy (Nikon Eclipse E200, Japan) in all tested bacterial isolates, which were later identified to the species level using Matrix-Assisted Laser Desorption/Ionization Mass Spectrometry (MALDI-TOF MS) using an ethanol-formic acid extraction procedure with an HCCA matrix solution according to the manufacturer’s instructions (Bruker Daltonik GmbH, Bremen, Germany) and Modrackova et al. [24].

### Data accessibility

Sequences of raw data files have been deposited in the NCBI database under Sequence Read Archive (SRA) accession numbers: SUB13811158 and BioProject ID: PRJNA1011505.

## Results

### Microbiome alpha and beta diversity

**Fig. 1:**
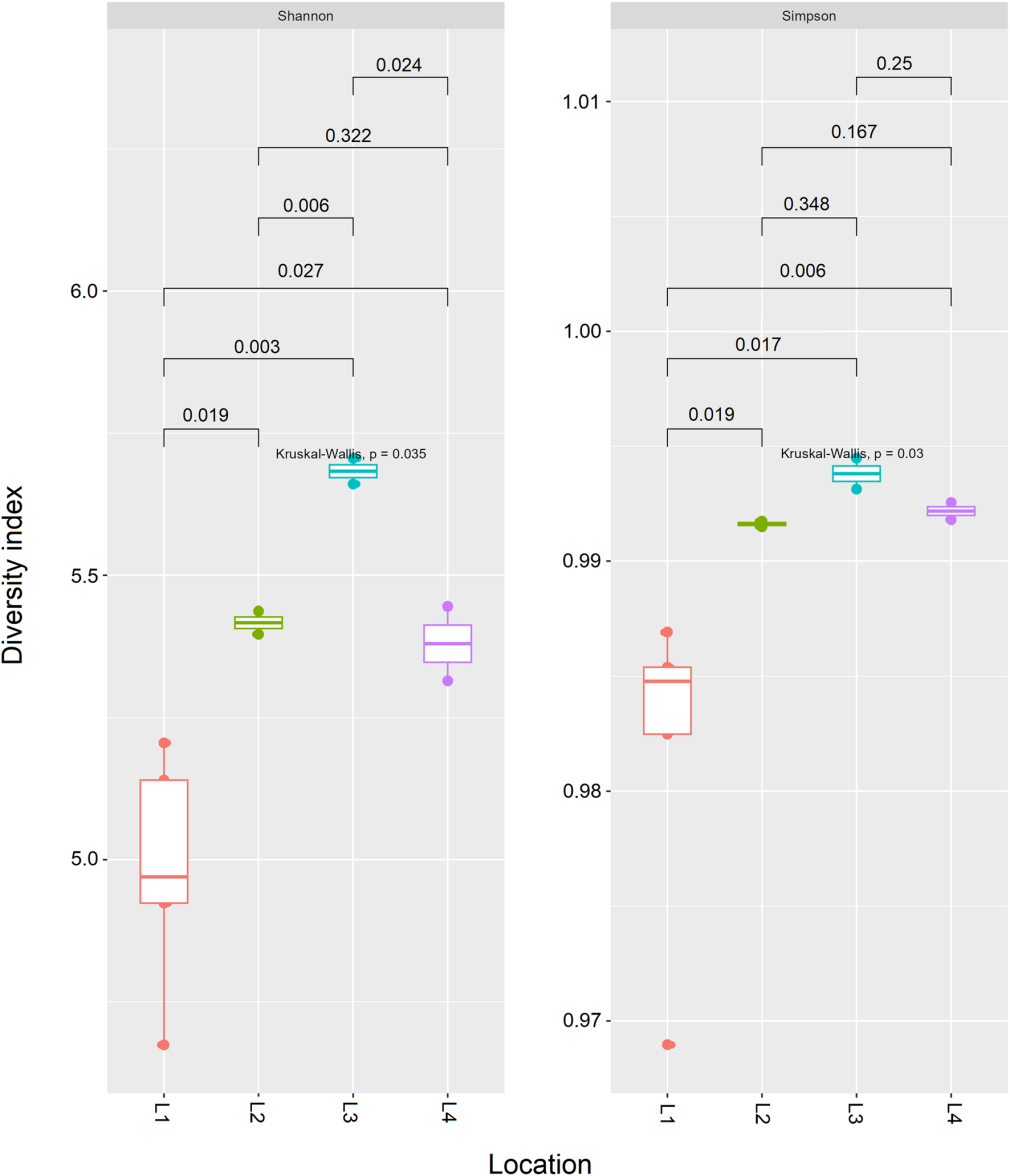
Microbiome diversity analysis of fecal samples of captive Southern Tamanduas from different locations. Boxplots illustrating alpha diversity using Shannon and Simpson diversity indices in bacterial community across different locations. P value ≤ 0.05 was considered statistically significant based on the Kruskal–Wallis test

In total, the microbiomes of eleven Southern Tamandua (*Tamandua tetradactyla*) from four different locations and captive conditions were analyzed. Three independent FS per each animal were used when DNA was isolated (Table 1). The results of alpha diversity using Shannon and Simpson diversity indexes are represented in the boxplot graph (Fig. 1). These differing values are reported in (Additional file 1: Table S1).

The highest bacterial diversity was observed in the gut microbiome of animals from location L3 using both Shannon and Simpson indexes. Using Simpson index, the diversity of gut microbiome of animals from location L3 was significantly higher in comparison to animals from location L1 (P = 0.017) but the difference was not significant in comparison to animals from locations L2 and L4 (P > 0.05). The diversity of gut microbiome of animals from location L1 was significantly low in comparison with the diversity of the gut microbiome of animals from other locations L2 (P = 0.019 in both indexes), L3 (P = 0.003 Shannon index, P = 0.017 Simpson index), and L4 (P = 0.027 Shannon index, P = 0.006 Simpson index). However, no significant difference was observed between animals from L2 and L4 locations using both indexes (P > 0.05).

**Fig. 2:**
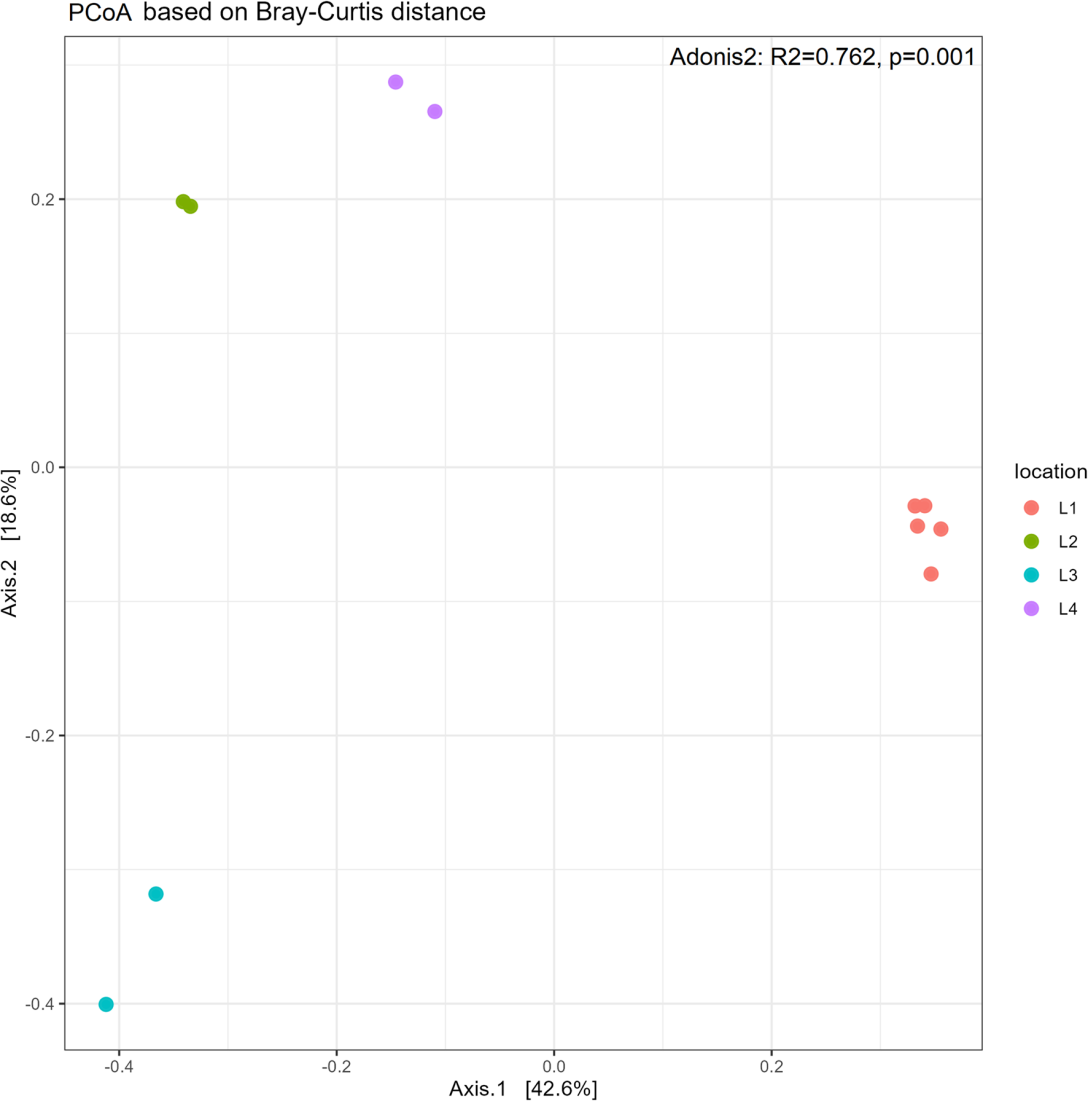
Microbiome diversity analysis of fecal samples of captive Southern Tamanduas from different locations. Beta diversity using Principal Coordinate Analysis (PCoA) plots based on the Bray Curtis distance showed distinct clusters of fecal microbiome diversity of animals from different locations. P value ≤ 0.05 was considered statistically significant

Principal coordinate analysis (PCoA) based on Bray–Curtis distance was performed to compare the diversity of the fecal microbiome of anteater at different locations (Fig. 2). Different clusters were distinguished and separated showing a significant difference in microbiome diversity (p = 0.001) between different locations. The samples of animals from location L1 were regrouped together and separated from the other groups while samples of animals from locations L2 and L4 were clustered closer.

### Microbiome composition

**Fig. 3:**
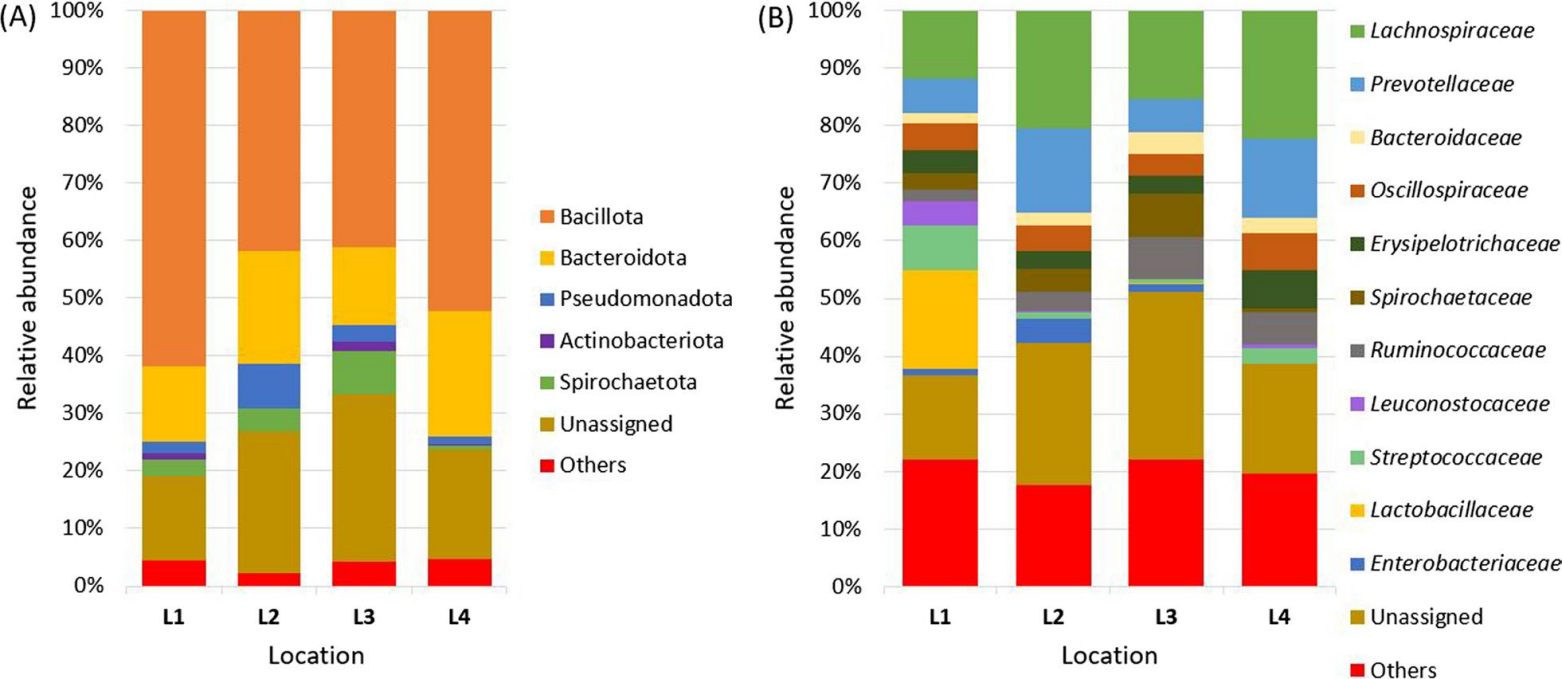
Relative abundance of bacterial populations at the phylum (**A**) and family (**B**) level in fecal samples of captive Southern Tamanduas from different locations. Taxa on the family level with a relative abundance of less than 3% are classified as “Others”

At the phylum level, the fecal microbiome of all animals was dominated by Bacillota (previously called Firmicutes) and Bacteroidota (previously Bacteroidetes) (Fig. 3A). Bacillota was the higher of the two in the fecal microbiome of animals from location L1. Spirochaetota (previously Spirochaetes) presented higher in the fecal microbiome of animals from location L3. Actinobacteriota (previously Actinobacteria) was less abundant in the fecal microbiome of animals from L2 and L4. Pseudomonadota (previously Proteobacteria) was detected less often, but in all groups, and mainly in L2.

At the taxonomic family level (Fig. 3B), *Lachnospiraceae* was highly abundant family in the fecal microbiome of animals from all four locations (from 11.74 to 22.33%). *Lactobacillaceae* (17.09%) was the most dominant bacterial family in the fecal microbiome of animals from location L1, in other locations, the representation was below 1%. Notably, the relative abundance of families *Streptococcaceae* (7.84%), *Leuconostocaceae* (4.24%)*,* and *Peptostreptococcaceae* (2.73%) was presented also higher in the fecal microbiome of animals from location L1 in comparison to animals from other locations. *Prevotellaceae* presented higher in the fecal microbiome of animals from locations L2 and L4 at percentages of 14.67% and 13.67% respectively. *Ruminococcaceae* (7.23%) and *Spirochaetaceae* (7.60%) both presented higher in the fecal microbiome of animals from location L3.

Microbiome composition at the phylum and family levels according to the animal samples are presented in Additional files 2 and 3: Figs. S1A and S1B.

**Fig. 4:**
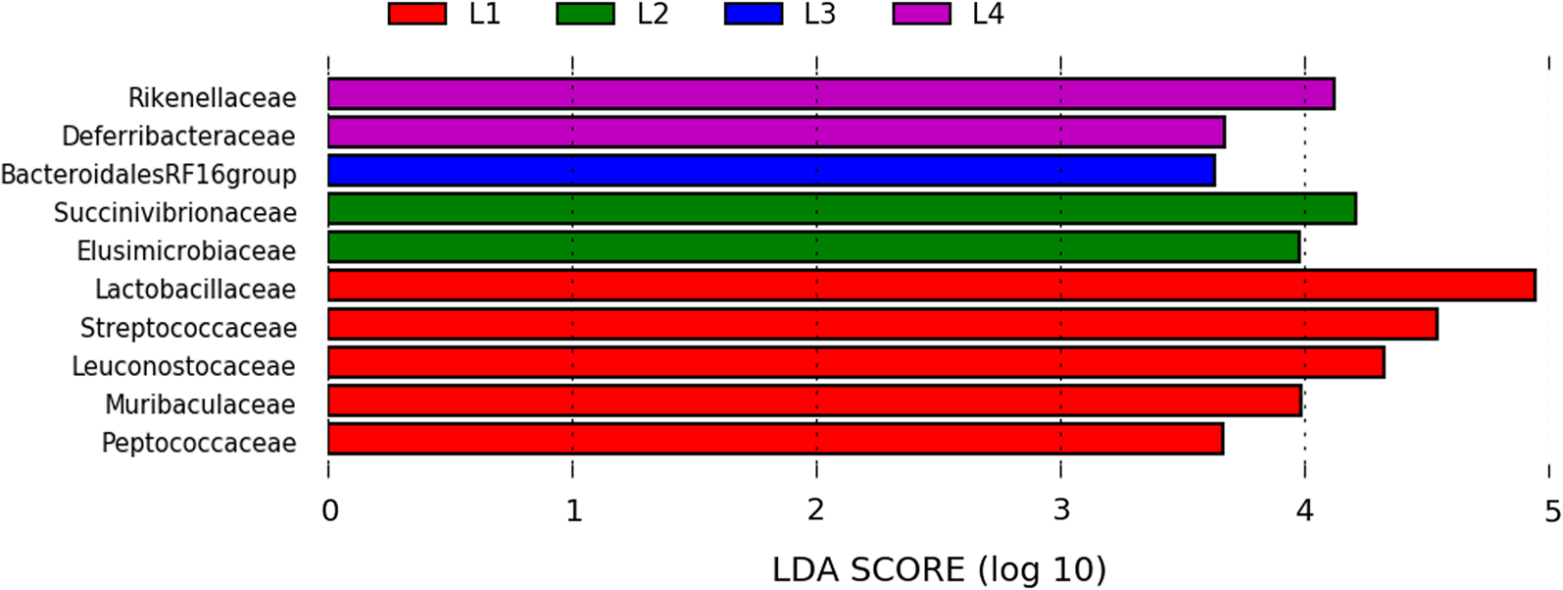
Linear discriminant analysis effect size (LEfSe) of taxa at taxonomic family level in fecal bacterial community of captive Southern Tamanduas from different locations with alpha values of 0.05 and a threshold value of 2.0

The linear discriminant analysis with effect size (LefSe) was used to detect bacterial families (biomarkers) with significant differential relative abundances in fecal microbiome of animals at different locations (Fig. 4). Five bacterial families were detected as biomarkers in the gut microbiome of animals from location L1. Two bacterial families in the fecal microbiome of animals from location L2 and L4. Only one biomarker was identified in the fecal microbiome of animals from location L3.

### Quantitative occurrence of cultured commensal groups

**Fig. 5:**
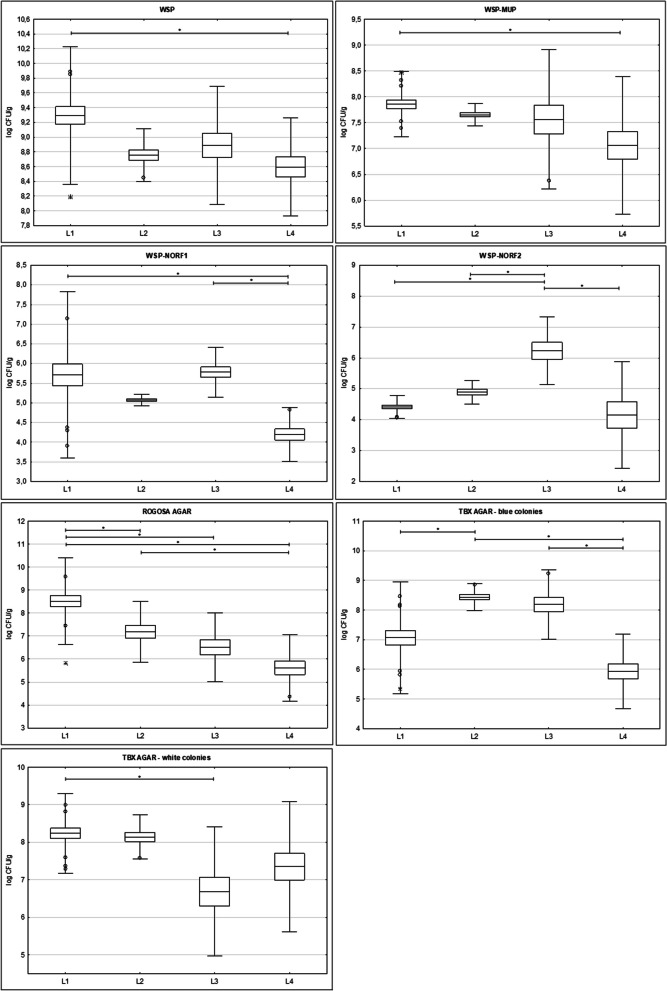
Cultivation counts of monitored bacterial groups

Non-selective and selective media were used to cultivate commensal groups of bacteria in FS of Southern Tamanduas collected from four locations in the Czech Republic with various captive conditions (Fig. 5). Average total counts of anaerobic and facultative anaerobic bacteria in FS of all anteater hosts ranged from 8.60 to 9.29 log CFU g^−1^. Southern Tamanduas’ different captive conditions correlated with various representations of detected cultivable bacterial groups. In general, total anaerobes were significantly higher for location L1 compared to L4. An equally significant trend was also detected for bacterial counts on WSP-MUP for L1 (7.86 ± 0.32 log CFU g^−1^) and L4 (7.06 ± 0.66 log CFU g^−1^). Enrichment of MUP-WSP agar with two different concentrations of norfloxacin (100 mg L^−1^ and 200 mg L^−1^) allowed for the detection of different numbers of target bacteria. Counts for L3 (5.78 ± 0.32 log CFU g^−1^) and L1 (5.71 ± 1.06 log CFU g^−1^) on WSP-NORF 1 were significantly higher compared to counts for L4 (4.20 ± 0.34 log CFU g^−1^), while counts on WSP-NORF 2 were significantly higher for L3 (6.23 ± 0.55 log CFU g^−1^) in comparison with all other monitored locations. Lactic acid bacteria on Rogosa agar were significantly higher in L1 (8.52 ± 0.95 log CFU g^−1^) compared to other locations, and in L2 (7.18 ± 0.66 log CFU g^−1^) in comparison with L4 (5.62 ± 0.72 log CFU g^−1^). Then, *E. coli* counts (blue colonies with β-glucuronidase activity) were higher in L2 (8.44 ± 0.22 log CFU g^−1^) compared to L1 (7.06 ± 0.94 log CFU g^−1^) and L4 (5.93 ± 0.63 log CFU g^−1^), higher were also in L3 (8.19 ± 0.59 log CFU g^−1^) compared to L4. Interestingly, the counts of remaining coliforms (white colonies) proved to be significantly higher in L1 (8.24 ± 0.53 log CFU g^−1^) compared to L3 (6.69 ± 0.86 log CFU g^−1^).

### Cultivable commensal bacterial groups in feces of Southern Tamanduas

More than one thousand grown isolates (*n* = 1078) were analyzed, and 92.58% of these isolates were successfully identified using MALDI-TOF MS at the species level, and 80 selected isolates were determined as unidentified.

In total, 63 bacterial species (Additional file 4: Table S2A) were isolated and identified on the used media designed for total anaerobes (WSP agar), bifidobacteria/clostridia (WSP-MUP, WSP-NORF 1, WSP-NOR 2 agars), lactic acid bacteria (Rogosa agar) and coliforms, as well as *E. coli* with β-glucoronidase activity (TBX agar). Each detected species was identified with a high degree of certainty with species consistency using MALDI-TOF MS. The most bacterial species were detected in animals from locations L1 and L2 (both, *n* = 35), L4 (*n* = 31), and least in L3 (*n* = 27). Table 2 presents the most frequently detected species (*n* = 32), selected from all detected species on the assumption that their occurrence was detected in two individual animals from two different locations. Moreover, *Citrobacter freundii*, *Cb. youngae*, *Clostridium baratii*, *C. colicanis*, *C. perfringens*, *Enterobacter cloacae*, *Eb. hormaechei*, *Enterococcus hirae*, *Escherichia coli*, *Pediococcus pentosaceus*, and *Streptococcus lutetiensis* were detected in feces of all animals. Also, orange-pigmented colonies of cocci identified as *Lactococcus garvieae* were frequently detected. The selective media supplemented with mupirocin and acetic acid, as well as with added norfloxacin allowed for mainly clostridial isolation. When *C. baratii*, *C. colicanis*, and *C. perfringens* were repeatedly detected in the FS of Southern Tamanduas from all locations. Whereas *Paeniclostridium sordellii* and *Paraclostridium bifermentans* were detected in animals from locations L1, L3, L4 (all zoos), but not in those from the private breeder (L2). The detection of bifidobacteria was rather random or rare. Only *Bifidobacterium animalis* was detected in multiple locations (L1 and L2; Table 2). Furthermore, *B. anseris* and *B. asteroides* (both, L4) were detected, and *B. pseudolongum* in FS of animals from location L1. Thus, the detected numbers on these media are more related to the quantification of viable culturable CFU of clostridia.

**Table 2:** The most frequently detected bacterial species of Southern Tamanduas by cultivation on selected media (presented species occurred in at least two animals from two different locations)

Detected species	L1 (AE1–5)	L2 (AE6–7)	L3 (AE8–9)	L4 (AE10–11)
*Actinomyces urogenitalis*	✖	✔	✔	✔
*Bacteroides fragilis*	✖	✖	✔	✔
*Bacteroides uniformis*	✖	✔	✔	✔
*Bifidobacterium animalis*	✔	✔	✖	✖
*Citrobacter braakii*	✖	✔	✖	✔
*Citrobacter freundii*	✔	✔	✔	✔
*Citrobacter youngae*	✔	✔	✔	✔
*Clostridium baratii*	✔	✔	✔	✔
*Clostridium colicanis*	✔	✔	✔	✔
*Clostridium perfringens*	✔	✔	✔	✔
*Collinsella aerofaciens*	✖	✔	✖	✔
*Enterobacter cloacae*	✔	✔	✔	✔
*Enterobacter hormaechei*	✔	✔	✔	✔
*Enterobacter kobei*	✔	✔	✖	✖
*Enterococcus faecalis*	✔	✔	✖	✔
*Enterococcus faecium*	✔	✖	✔	✔
*Enterococcus hirae*	✔	✔	✔	✔
*Escherichia coli*	✔	✔	✔	✔
*Klebsiella pneumoniae*	✔	✔	✖	✖
*Lactobacillus curvatus*	✔	✖	✔	✖
*Lactococcus garvieae*	✔	✔	✖	✔
*Lactococcus lactis*	✔	✖	✖	✔
*Limosilactobacillus reuteri*	✔	✖	✔	✖
*Paeniclostridium sordellii*	✔	✖	✔	✔
*Parabacteroides distasonis*	✖	✔	✔	✖
*Paraclostridium bifermentans*	✔	✖	✔	✔
*Pediococcus acidilactici*	✖	✖	✔	✔
*Pediococcus pentosaceus*	✔	✔	✔	✔
*Phocaeicola massiliensis*	✖	✖	✔	✔
*Phocaeicola vulgatus*	✖	✔	✖	✔
*Streptococcus lutetiensis*	✔	✔	✔	✔
*Weissella paramesenteroides*	✔	✔	✔	✖

L—location, AE—animal host (anteater), ✔—detected, ✖—not detected, the exact location and animal host data are presented in Table 1

The elective agar (WSP) as well as selective media used for cultivation of bifidobacteria/clostridia also allowed for the growth of enterococci, pediococci, streptococci, and other bacteria (Additional file 4: Table S2B). Detection of lactobacilli was paradoxically more frequent on these media than on Rogosa agar, where *Pc. pentosaceus* dominated, and frequently occurred *Weissella paramesenteroides* (Additional file 4: Table S2C). On the TBX medium, it was mainly the genera such as *Escherichia*, *Enterobacter*, *Enterococcus*, *Citrobacter*, *Klebsiella* that were detected, along with some bacteria that were not coliforms e.g*. Lc. garvieae*, *Pc. pentosaceus*, *Strep. lutetiensis*, and others less frequently occurred. *E. coli* with β-glucoronidase activity were detected in FS of all Southern Tamanduas, but also white colonies of *E. coli* were detected in nine out of the eleven animals (Additional file 4: Table 2D).

Some species were detected based on location. These were, for example, species such as *Enterococcus casseliflavus*, *Hungateli hathewayi*, *Phocaeicola massiliensis*, *P. vulgatus*, *Prevotella copri*, *Raoultella ornithinolytica*, *R. planticola*, *Secundilactobacillus malefermentans*, *Staphylococcus capitis*, and *S. warneri* that were detected only with the private breeder (L2), then *Sphingobacterium thalpophilum*, *Veillonella dispar*, *V. ratti*, and *Weissella cibaria* (L1), *W. confusa* (L4), and *Acinetobacter lwoffii *(L3). On the other hand, *Actinomyces urogenitalis* was also detected in animals from three locations (L2, L3, L4).

## Discussion

### Fecal microbiome of captive myrmecophagous represented by Southern Tamanduas

Microbiome studies on several animal species were performed during the last decade through the rapid development of DNA sequencing methods and tools. This advance makes it possible to analyze hundreds of samples from different animal species simultaneously to obtain a general overview of their microbiome. However, there is still uncertainty as to the variability of the microbiome of different animal orders and whether certain bacteria within a species are subject to greater fluctuations than others [25]. The first research addressing the microbiome of myrmecophages was published by Delsuc et al. [2], who reveal specialized placental myrmecophages as a spectacular case of large-scale convergence in gut microbiome composition. Later, the study of de Jonge et al. [12] described similarities and differences in the gut microbiome composition in 54 animal specimens across 42 species, including the Giant Anteater. Using non-metric, multi-dimensional scaling analysis based on Bray–Curtis distances, the primarily herbivoric orders Perissodactyla, Proboscidae, Diprodontia, Artiodactyla, and Pilosa were somewhat clustered together, while Carnivora, Rodentia, Lagomorpha, and Primates were grouped separately. The highest richness in alpha diversity was seen in the order Pilosa, represented by the Giant Anteater in comparison with other captive animals. Here we present a dataset that includes the fecal microbiome of captive Southern Tamanduas from varying breeding and diet conditions. The fecal microbiome of analyzed captive animals was dominated by the phyla Bacillota (Firmicutes) and Bacteroidota (Bacteroidetes). Pseudomonadota (Proteobacteria), Spirochaetota (Spirochaetes), and Actinobacteriota (Actinobacteria) were less abundant, and their abundance was more varied based on the location and animal. Similar results to the phyla results were presented by Delsuc et al. [2]. At the taxonomic family level, *Lachnospiraceae, Prevotellaceae, Bacteroidaceae, Oscillospiraceae, Erysipelotrichaceae, Spirochaetaceae, Ruminococcaceae, Leuconostocaceae*, *Streptococcaceae,* and *Peptostreptococcaceae* were represented in the fecal microbiome of animals from all locations. *Lactobacillaceae* dominated in L1. Other locations yielded low abundance or no representation at all. On the other hand, the absence and high representation of *Lactobacillaceae* may be primarily related to diet. Only animals from location L1 had a zoo-made diet, while others from locations L2–L4 had a complete commercial diet. The presence of *Lactobacillaceae* taxa in the gut microbiome is highly dependent on the host's diet [26, 27].

### The effect of diet and breading conditions on the gut microbiome

Studies examining diverse arrays of animal species and comparing the microbiome of different habitats/groups and species has provided valuable insights [10, 22, 28–32].

We analyzed microbime composition and selected cultivable microbes from 33 FS of 11 anteaters (*Tamandua tetradactyla*) from 4 locations. The diet in these three locations (L2–L4) was mainly a complete commercial diet and in location L1 a zoo-made diet. This difference can explain the significantly different microbial profile of the L1 animal group, as it had already been described in regard to other animal species and humans [8, 33]. Moreover, the use of probiotic and prebiotic intervention can cause microbial shifts [34, 35]. Namely, fructooligosaccharides and inulin were present in the complete commercial diet, as well as the probiotic strain of *Enterococcus faecium* E1708. However, species *Ec. faecium* was also present in other probiotic supplements, therefore, it was supplied to all animals through their diet. A more varied probiotic supplement (*Saccharomyces cerevisiae* CNCM I-1077, *Bacillus licheniformis* DSM 5749, *Bacillus subtilis* DSM 5750, and *Enterococcus faecium* NCIMB 10415) was administered to the L1 group, which may explain the differing microbiome profile for example through microbial interaction. Detection of mentioned species through cultivation screening can indicate their transit through the gastrointestinal tract of anteaters. However, we are not able to distinguish whether these were the administered probiotic strains or the original strains of Southern Tamanduas. However, to increase the probability of colonization, it is recommended to use a strain originally isolated from the digestive tract of the species for which the given probiotics are intended [36]. In addition, the used insects as feed could potentially be a source of microbes and influence the composition of the fecal microbiome [37]. Just like the diet and other supplements, the presence of another animal species during exposure could potentially be the source of the differing microbiome, especially during longer-term exposure [38]. All anteaters from zoo locations shared exposure with other animal species while animals from the private breeder did not. Animal species-specific responses within the microbiome might play a pivotal role in determining which animals can better adapt to changes brought about by captivity, where analyses of the intestinal microbiome might have a significant role [39].

All fecal samples used in this study were collected from animals in captive conditions. Animals in captivity experience a range of altered conditions compared to their wild counterparts, including changes in diet, social structure, population density, human contact, medical treatment, and antibiotic administration [40]. These changes introduce selective pressures on the animals, affecting their gut microbiome composition, and reducing diversity and functional aspects of the microbiome [10, 41]. These alterations in the gut microbiome can potentially lead to negative effects on the health of the animals [39, 40]. It is essential to deepen our understanding of the effects of captivity on animal microbiomes in order to provide optimal care to create more suitable captive conditions that align with the animal's natural biology and wellbeing, particularly in terms of its health and overall welfare [10, 39, 42, 43].

### Species variability and significance of anteater cultivable microbes

High proportions of bacteria are culturable across major biomes within known techniques [44] when the gut microbiota is an infinite source of microbes and is considered one of the key elements that reflect the health status of the host [45]. Here we focused on well-cultivated commensal microbes to see their species variability across captive anteater hosts. Although the family *Clostridiaceae* was not among the most represented families, its presence was common for the microbiome of all anteaters. It corresponded with the cultivation results, where clostridia dominated among viable anaerobes on mupirocin and norfloxacin media with relatively stable species representation in comparison with bifidobacteria. Clostridia seem to be naturally resistant to mupirocin and norfloxacin, and low or absent quantitative representation of bifidobacteria in the fecal sample allows their detection [46, 47]. The presence of *C. baratii*, *C. colicanis*, *C. perfringens, Paenicl. sordellii*, and *Paracl. bifermentans* in animal feces or farm manure was documented [48], some of them pose a pathogenic risk [49], however due to their metabolic activity, they can play an essential role in anteater digestion. *Clostridium* species can utilize large amounts of nutrients that cannot be digested by the host and produce lots of short-chain fatty acids, which play a noticeable role in intestinal homeostasis [50]. Bifidobacteria are considered a beneficial microbial group and their occurrence is being researched across different animal species [51]. The presence of *Bifidobacterium* spp. in anteater feces was in unstable counts and they were not the dominant cultivable bacterial group, as well as *Bifidobacteriaceae* family was not present among mainly occurred families in the fecal microbiome of captive Southern Tamanduas. Bifidobacterial species occurrence varied across animals and locations. Contact with other animal species and the presence of insects as feed or insect-based products in the anteater’s diet could potentially be the source of detected bifidobacteria [22, 37, 52, 53]. Including suitable prebiotic substrates with a bifidogenic effect in the diet of anteaters can be an option to increase their quantitative representation to support beneficial effects [54], because captivity and associated dietary changes, including missing natural prebiotics substrates [55], may be a factor for the low presence of bifidobacteria in the anteaters in this study. Similarly, the cultivable lactobacilli occurrence was variable and low, compared to other lactic acid bacteria such as *Weissella* spp. and pediococci, mainly *Pc. pentosaceus*. Interestingly, lactobacilli [56] and *Pediococcus* spp. can have many beneficial roles covering aspects such as immunomodulation [57], growth enhancement in farm animals [58, 59], and resistance to infection [60]. Another frequently detected coccus in the feces of all anteaters was *Strep. lutetiensis*, its role in the intestinal microbiota is somewhat unclear. However, published works indicate a possible pathogenicity [61, 62]. A zoonotic potential is also carried by *Lc. garvieae*. Granadaene-producing colonies were commonly detected in anteater feces and their presence may be related to insects as feed in the diet of captive anteaters [37]. Coliforms belong to common commensals of the gut microbiota, but they include opportunistic pathogens as well [63]. TBX medium is a selective, chromogenic medium for the detection and enumeration of *E. coli* in food products and animal feed, however, it seems to be suitable for detection of coliforms in FS [64, 65]. In this study, the medium allowed us to quantify both *E. coli* and other taxa of coliform bacteria such as *Enterobacter* spp., *Citrobacter* spp., and *Klebsiella* spp. in the anteater microbiota. The mentioned groups were quantified in relatively constant numbers and species variability. The TBX medium was not selective for enterococci, therefore, their presence is considered in the presented numbers of other coliforms. Interestingly, a fecal metagenomics study of Malayan pangolins [66] found several species that likely play roles in the digestion of cellulose and may be able to degrade chitin; including *Enterobacter cloacae*, *Lactococcus lactis*, and *Klebsiella pneumoniae*, which we detected as well as common Southern Tamanduas commensals. Also, Cheng et al. [13] demonstrated the complex and diverse interactions between hosts such as anteaters, echidnas, and pangolins (of the orders Pilosa, Monotremata, and Pholidota, respectively) and their symbiotic microbiota that have provided adaptive solutions for nutritional and detoxification challenges. As well as spatially complementary cooperation involved in the degradation of ants’ and termites’ chitin exoskeletons was revealed. Their study contributes new insights into the dietary evolution of mammals and the mechanisms involved in the coordination of physiological functions by animal hosts and their gut commensals.

It can therefore be assumed that metabolic functions of bacterial commensals of anteaters are associated with the ability to use specific substrates found in the diet of these hosts.

## Conclusion

Using application sequencing, the most represented taxa in the fecal microbiome of captive Southern Tamanduas from four locations in the Czech Republic were determined at the phylum and family levels. The microbiome analysis in which anteater locations were considered, showed that the captive conditions significantly influenced the microbiome profile of the analyzed animal FS. The cultivation analysis focused on anaerobic and facultative anaerobic bacteria, especially bifidobacteria and clostridia, lactic acid bacteria, and coliforms, detected culturable microbes and emphasized typical bacterial species present in the microbiota of captive Southern Tamanduas. These findings can help optimize breeding and dietary conditions and prevent possible microbial infections connected to fecal microbes.

### Supplementary Information

**Additional file 1. **Values supporting results of alpha diversity using Shannon and Simpson diversity (Table S1).

**Additional file 2. **Microbiome composition at the phylum level according to the animal samples (Fig. S1A).

**Additional file 3. **Microbiome composition at the family level according to the animal samples (Fig. S1B).

**Additional file 4. **Detected counts and species variability across the animal samples using culture-dependent methods; all detected bacterial species (Table S2A), results based on the media: different Willkins agars (Table S2B), Rogosa agar (Table S2C), TBX agar (Table S2D).
