# Supplementary figures and images for: Microbiome composition and presence of cultivable commensal groups of Southern Tamanduas (Tamandua tetradactyla) varies with captive conditions
Source: Anim Microbiome. 2024 May 2;6:21. doi: 10.1186/s42523-024-00311-w (PMC11064412; doi:10.1186/s42523-024-00311-w)

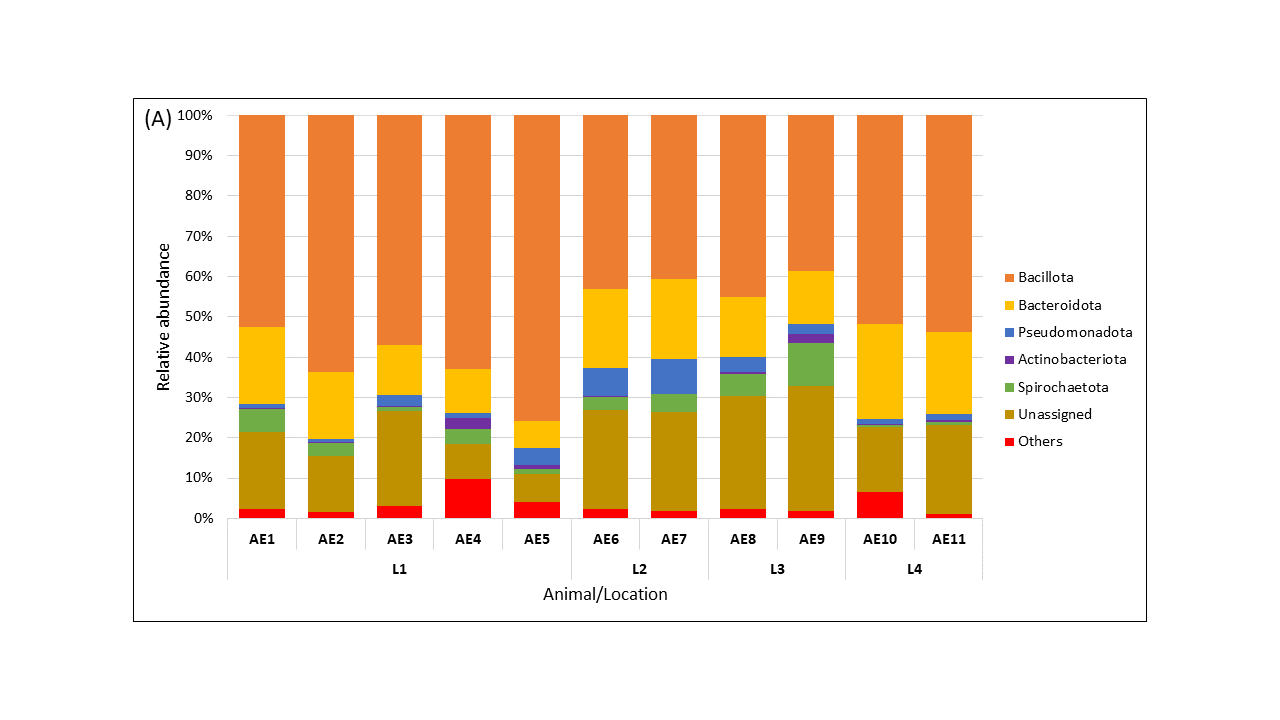

Supplement: Supplementary file 2 — Additional file 2. Microbiome composition at the phylum level according to the animal samples (Fig. S1A). [file 42523_2024_311_MOESM2_ESM.png]

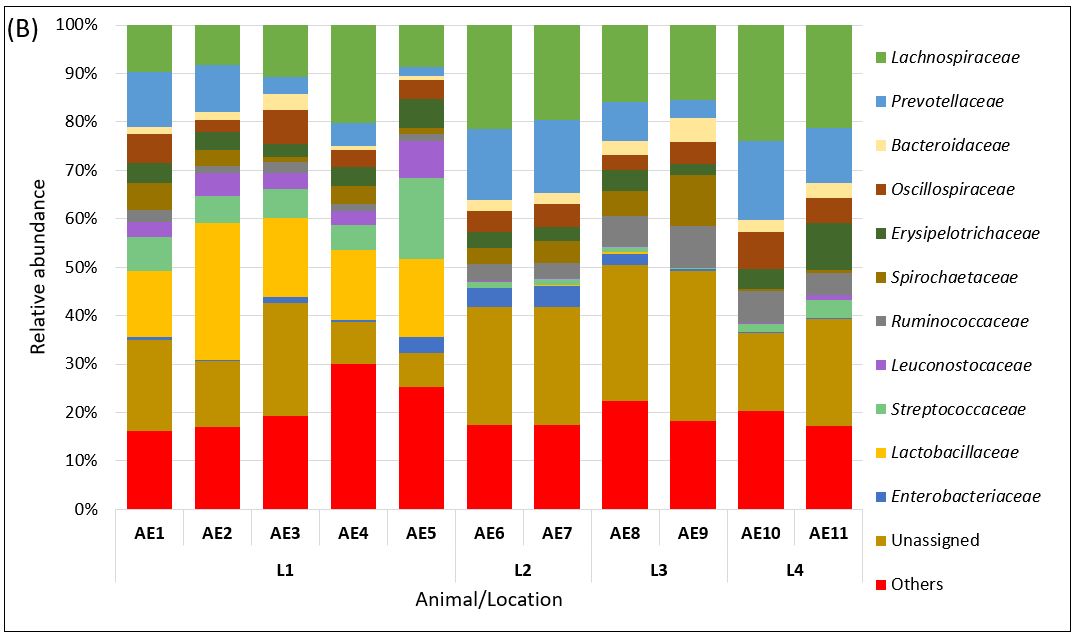

Supplement: Supplementary file 3 — Additional file 3. Microbiome composition at the family level according to the animal samples (Fig. S1B). [file 42523_2024_311_MOESM3_ESM.jpg]
